# Water-Soluble Humic Materials Modulating Metabolism and Triggering Stress Defense in Sinorhizobium fredii

**DOI:** 10.1128/spectrum.00293-21

**Published:** 2021-08-25

**Authors:** Xiaoqian Qiu, Tongguo Gao, Jinshui Yang, Entao Wang, Liang Liu, Hongli Yuan

**Affiliations:** a State Key Laboratory of Agrobiotechnology and Key Laboratory of Soil Microbiology, Ministry of Agriculture, College of Biological Sciences, China Agricultural University, Beijing, China; b College of Life Sciences, Hebei Agricultural University, Baoding, China; c Departamento de Microbiología, Escuela Nacional de Ciencias Biológicas, Instituto Politécnico Nacional, Mexico City, Mexico; USDA - San Joaquin Valley Agricultural Sciences Center

**Keywords:** water-soluble humic materials, RNA-Seq, metabolism, stress defense, *Sinorhizobium fredii*

## Abstract

Bacteria have evolved a series of mechanisms to maintain their survival and reproduction in changeable and stressful environments. In-depth understanding of these mechanisms can allow for better developing and utilizing of bacteria with various biological functions. In this study, we found that water-soluble humic materials (WSHM), a well-known environment-friendly plant growth biostimulant, significantly promoted the free-living growth and survival of Sinorhizobium fredii CCBAU45436 in a bell-shaped, dose-dependent manner, along with more-efficient carbon source consumption and relief of medium acidification. By using RNA-Seq analysis, a total of 1,136 genes significantly up-/downregulated by external addition of WSHM were identified under test conditions. These differentially expressed genes (DEGs) were enriched in functional categories related to carbon/nitrogen metabolism, cellular stress response, and genetic information processing. Further protein-protein interaction (PPI) network analysis and reverse genetic engineering indicated that WSHM might reprogram the transcriptome through inhibiting the expression of key hub gene *rsh*, which encodes a bifunctional enzyme catalyzing synthesis and hydrolysis of the “magic spot” (p)ppGpp. In addition, the root colonization and viability in soil of S. fredii CCBAU45436 were increased by WSHM. These findings provide us with new insights into how WSHM benefit bacterial adaptations and demonstrate great application value to be a unique inoculant additive.

**IMPORTANCE** Sinorhizobium fredii CCBAU45436 is a highly effective, fast-growing rhizobium that can establish symbiosis with multiple soybean cultivars. However, it is difficult to maintain the high-density effective viable cells in the rhizobial inoculant for the stressful conditions during production, storage, transport, and application. Here, we showed that WSHM greatly increased the viable cells of S. fredii CCBAU45436 in culture, modulating metabolism and triggering stress defense. The root colonization and viability in soil of *S. fredii* CCBAU45436 were also increased by WSHM. Our results shed new insights into the effects of WSHM on bacteria and the importance of metabolism and stress defense during the bacteria’s whole life. In addition, the functional mechanism of WSHM may provide candidate genes for improving environmental adaptability and application potential of bacteria through genetic engineering.

## INTRODUCTION

In nature, various stress conditions such as nutrient deprivation, high-temperature, low pH, drought, and salinity inhibit the growth and survival of bacteria. Under laboratory conditions, bacteria will enter stationary phase, in which cells divide slowly or stop growth, due to unfavorable factors such as nutrient starvation, accumulation of toxic by-products, unsuitable pH, and osmotic stress (1, 2). To ensure growth and survival under harsh environments, bacteria need to adapt their life functions, including energy metabolism, growth control, and stress responses, and the regulatory integration of these processes (3). In-depth understanding of these stress-adaptation mechanisms of bacteria is important for better development and utilization of bacterial functions.

In the past few decades, the complex stress response network has been gradually elucidated by integrating the “stressomics” approaches and different induced conditions or genetic mutants. These responses include protecting the cell envelope, regulating cellular metabolism, and defending macromolecules, etc. (4, 5). The cell envelope is the first barrier of bacteria; when cells are confronted with environmental perturbations, the envelope stress responses (ESRs), including Cpx and σ^E^ system, are trigged to maintain cellular homeostasis (6, 7). Bacteria adjust their metabolism when undergoing stress. For example, Christodoulou et al. (8) found that pentose phosphate (PP) pathway flux increases instantly to stabilize cellular NADPH levels for oxidative stress defense when Escherichia coli is exposed to H_2_O_2_. Any stress leads to protein denaturation and influences the growth of bacteria, and then a set of chaperones and proteases are induced to maintain protein homeostasis and help bacteria overcome stresses (9, 10).

The alarmone guanosine tetra- or pentaphosphate [(p)ppGpp] is considered to be one of the key regulators and drivers for cell growth and stress resistance (3, 11). As a governor of global resource allocation, (p)ppGpp can rearrange the cellular investment to cope with a constantly changing environment (12, 13). Zhu and Dai (14) reported that both increase and decrease of (p)ppGpp levels inhibited the growth of bacteria significantly. In addition, the cellular (p)ppGpp levels were elevated upon heat shock, salt stress, and oxidative stress, and a (p)ppGpp^0^ strain showed more sensitivity to stress, indicating that (p)ppGpp improved the stress tolerance of bacteria (15). The concentrations of intracellular (p)ppGpp are controlled by a highly conserved and widely distributed protein family, RelA-SpoT homologs (RSH), which contain N-terminal synthetase and/or hydrolase domains together with two conserved regulatory domains at the C terminus (16). In alphaproteobacteria, (p)ppGpp concentration is regulated by a single RSH protein, which has bifunctional activities of synthesis and hydrolysis (17).

However, previous studies are often limited to a specific stress, which leads to decreases in cell activity and yield and makes it difficult to understand the cooperative relationship of various stress responses (18). By studying the response of bacteria to a positive stimulant, the mechanism of promoting bacterial growth can be revealed more directly and accurately so as to better employ the beneficial bacteria. Water-soluble humic materials (WSHM) are active organic substances composed of crosslinked alkyl or aromatic groups and contain many functional groups, such as carboxyl and carbonyl groups, etc. (19). It has been widely accepted that WSHM manifest beneficial activity to stimulate plant growth and enhance stress resistance of plants in an eco-friendly way (20). Moreover, WSHM function as biostimulants to increase the cell density of rhizobia in culture, induce the *nod* gene expression, and enhance the efficiency of nitrogen fixation. The cell density of Bradyrhizobium liaoningense and Sinorhizobium meliloti in medium supplied with 0.5 g/liter WSHM was about 8 or 14.8 times higher than that of the control, respectively (21, 22). Rhizobia can invade the plant and interact symbiotically with the legume, so the application of high-efficiency rhizobial inoculants has proven to be a strategy to improve the yield and quality of legumes. However, the stressful conditions during production, storage, transport, and application make it still a challenge to maintain the high density of effective viable cells in the inoculant. In the present study, we found that WSHM promoted growth and survival of *S. fredii* CCBAU45436, a highly effective, fast-growing rhizobium that is able to establish a symbolic relationship with multiple soybean cultivars, for which the maximum count of viable cells was ultimately increased 1,428 times that of the control. To our best knowledge, it has not been reported that the number of viable cells in batch culture increased significantly by adding a dash of certain substances. A comprehensive analysis to understand the regulatory mechanism of WSHM is of great benefit to its application and may provide clues for improving the environmental adaptability and application potential of bacteria through genetic engineering.

## RESULTS AND DISCUSSION

### Promotion of rhizobial growth and survival by WSHM.

**FIG 1:**
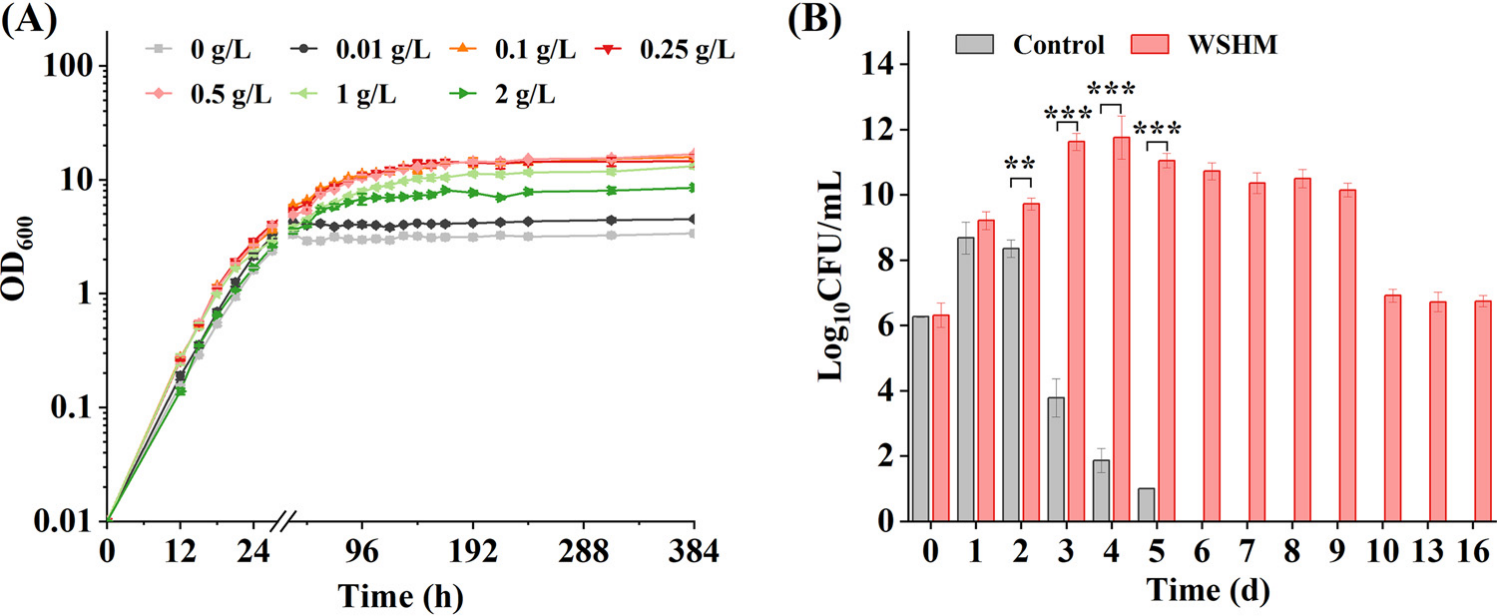
Effects of WSHM on the growth and survival of *S. fredii* CCBAU45436 in YM broth. (A) Growth curves of *S. fredii* CCBAU45436 in YM broth supplied with different concentrations of WSHM. (B) Effects of 0.5 g/liter WSHM on the number of viable cells. The data are presented as the means ± standard deviations from triplicate experiments. (*t* test: *, *P < *0.05; **, *P < *0.01; ***, *P < *0.001).

The growth of test strain *S. fredii* CCBAU45436 in yeast mannitol (YM) broth was promoted by WSHM, but the efficiency varied according to the concentration. The concentrations of 0.1 to 0.5 g/liter WSHM showed the best promoting effect, with faster exponential growth rates and the final cell concentration (represented by optical density at 600 nm [OD_600_]) increased more than 3-fold (*t* test, *P < *0.001). The promotion effect of lower concentration (0.01 g/liter) or higher concentration (2 g/liter) is slightly inferior (Fig. 1A). The concentration of 0.5 g/liter was selected for further study about the effect of WSHM on the viability of *S. fredii* CCBAU45436. The results showed that supplying WSHM in YM broth significantly promoted the growth and survival of *S. fredii* CCBAU45436 (Fig. 1B). The strain cultured in YM broth reached the maximal viable number of 6.77 × 10^8^ CFU/ml in 24 h, and then cells died rapidly after 48 h and decreased to 10 CFU/ml after 120 h. In treatment of 0.5 g/liter WSHM, *S. fredii* CCBAU45436 continued to grow exponentially for 72 h and produced a maximal population of 9.67 × 10^11^ CFU/ml after 96 h, which was 1,428-fold higher than that of the control (*t* test data transformation, *P < *0.01). Moreover, WSHM lengthened the survival time of *S. fredii* CCBAU45436, and the viable cells maintained above 10^10^ CFU/ml up to 216 h and 5.81 × 10^6^ CFU/ml after 384 h. These results indicate that appropriate concentration of WSHM significantly promoted the growth and survival of rhizobia. The rhizobial growth-promoting properties were consistent with those observed in the previous studies, although the degrees of promotion were different (21, 22).

The number of viable rhizobia is one of the most important factors for evaluating inoculant quality (23). Currently, polymers such as alginate, pectin, and chitosan may be superior additives; however, the production of these polymers from raw materials requires pretreatment and high costs (24). Liquid formulations typically contain oil or polymer, in which the water-in-oil emulsions could improve the viability of rhizobia by reducing water evaporation (25), but they could not increase the cell density of rhizobia. Compared to the most commonly used solid carrier peat, the porous structure and rich elements of WSHM can provide a favorable habitat for bacteria proliferation and survival (26). Moreover, WSHM are easily soluble in water, so they can also be used as a good additive for liquid formulations. Particularly, WSHM are beneficial to numerous microbial species such as Acinetobacter junii and rhizobia (27), while they can be applied directly to plants to improve their nutrient uptake, growth, and resistance to stress (26, 28, 29). The bipromoting effects of WSHM on bacteria and plants make them unique, high-efficiency inoculants additive to increase bacterial density, extend shelf life, and stimulate plant growth.

### Carbon source utilization efficiency improved by WSHM.

**FIG 2:**
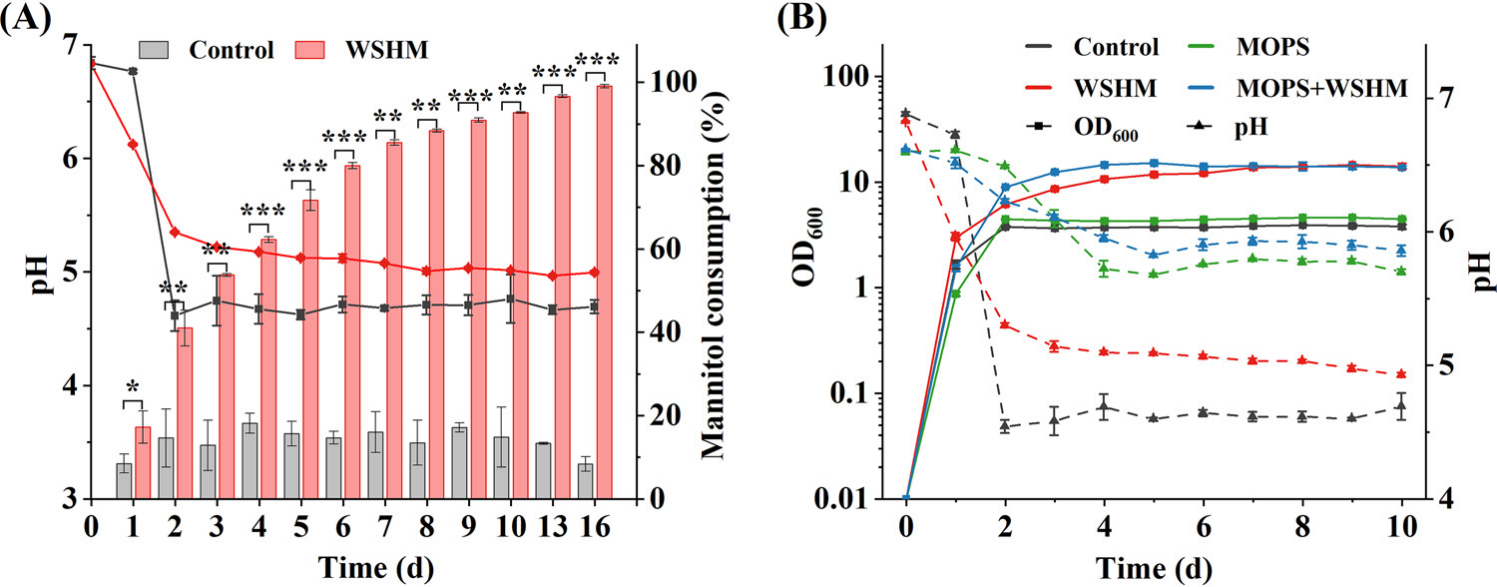
Effects of WSHM on carbon source consumption, pH values, and growth of *S. fredii* CCBAU45436. (A) Effects of WSHM on mannitol consumption and pH. (B) Effects of WSHM on growth and pH in YM broth with/without MOPS buffer. The data are presented as the means ± standard deviations from triplicate experiments. (*t* test: *, *P < *0.05; **, *P < *0.01; ***, *P < *0.001).

In order to reveal the reasons why WSHM promoted the growth of *S. fredii* CCBAU45436, we examined whether WSHM was used directly as a carbon or nitrogen source. The results showed that neither external addition of mannitol nor that of NH_4_Cl with an equivalent amount of carbon or nitrogen in 0.5 g/liter WSHM could significantly promote the growth of rhizobia in YM broth (Fig. S1). This suggested that WSHM may promote the growth of rhizobia by “hormone-like” function. Next, we analyzed the consumption of mannitol to test whether WSHM promoted the utilization of a main carbon source in YM broth. The result showed that the mannitol consumption by *S. fredii* CCBAU45436 was significantly increased by the addition of 0.5 g/liter WSHM (8.5% versus 17.2% on the 1st day, 14.6% versus 41.0% on the 2nd day; Fig. 2A). In addition, the mannitol consumption increased until exhausted, coupling with the continuous growth and long-term survival of rhizobia in treatment of 0.5 g/liter WSHM, while the mannitol was not further consumed in the control as the cells entered the decline phase. Accordingly, the mannitol consumption showed a positive correlation with OD_600_ (Pearson’s *r* = 0.995, *P < *0.001) (Fig. S2).

### Medium acidification alleviated by WSHM.

The above results indicate that carbon source was not a limitation for growth of *S. fredii* CCBAU45436 in YM broth. Considering that changes of pH at a later stage of growth might be one of the important factors affecting bacterial growth (2), pH values of the cultures were monitored. The results showed that the pH decreased rapidly along with the growth of *S. fredii* CCBAU45436 in YM broth and eventually stabilized at around 4.6, while the pH of culture supplied with WSHM was 5.35 at 48 h and decreased slowly to about 5.0 (Fig. 2A). These results suggest that the promotion effect of WSHM on the growth and survival of rhizobia should be partly attributed to its slowing down of pH decline in the culture. For most *Sinorhizobium* strains, the optimal pH for growth is near neutral (30). Hellweg et al. (31) found that S. meliloti 1021 was extremely sensitive to acid: although its growth curve above pH 6.0 was not significantly different from that at pH 7.0, it showed a reduced growth rate at pH 5.75 and was not able to grow at pH 5.5. However, the difference of pH changes between the control and WSHM treatment was not enough to explain the promotion effect of WSHM, because only a weak promotion on the growth of *S. fredii* CCBAU45436 was observed in YM broth buffered with morpholinepropanesulfonic acid (MOPS; pH 5.7) (Fig. 2B). In addition, no significant difference was observed in the ultimate cell density of *S. fredii* CCBAU45436 between the YM broth supplied with WSHM buffered by MOPS or not. These results suggested that a more fundamental reason for the promotion of WSHM might be that it enhanced the resistance of rhizobia to the stress.

### Genomic mechanism of WSHM improving rhizobial growth and survival.

**Transcriptomic profiles general overview.** The above results show that WSHM act as biostimulants to improve the growth and survival of *S. fredii* CCBAU45436. To reveal candidate genes involved in the response to WSHM, we performed a comparative transcriptome analysis on the cells cultured in YM broth with or without WSHM at 24 h, which is the key point of physiological differentiation from the logarithmic growth phase to the stationary phase. A total of 98,983,378 clean reads were obtained, and 95.57% of them were uniquely mapped to the reference genome (Table S1). Among the 6,752 annotated genes, expressions of 1,136 (16.8%) were significantly different, of which 716 were upregulated and 420 were downregulated by addition of WSHM. In *S. fredii* CCBAU45436 genome, a chromosome (cSF45436, 4.16 Mb), a chromid (pSF45436b, 1.96 Mb), a symbiotic plasmid (pSF45436a, 0.42 Mb), and two accessory plasmids (pSF45436d with 0.20 Mb and pSF45436e with 0.17 Mb) were identified (32). Among these replicons, DEGs were significantly enriched on the chromosome and pSF45436d. A total of 332 genes of the chromosome were significantly downregulated, accounting for 79.05% of the total downregulated genes, while 103 (50.74%) of the 203 genes on pSF45436d were significantly upregulated (Fig. S3; Table S2). The enrichment of downregulated genes on the chromosome suggests that the core function may be downregulated by WSHM, as core functions such as clusters of orthologous groups (COG) classes J (translation, ribosomal structure and biogenesis), A (RNA processing and modification), and Z (cytoskeleton) were usually enriched on chromosome (33). Although it is unclear whether pSF45436d plays a special role in *S. fredii* CCBAU45436, a large number of genes in this plasmid are annotated as stress response genes, suggesting that pSF45436d may be involved in adaptation to environmental stress, as revealed earlier (34). Gao et al. (21) reported that WSHM were effective in inducing the *nod* gene expression in *B. liaoningense*; however, the expression of key symbiosis genes such as *nod*/*nif*/*fix* was not affected by WSHM in *S. fredii* CCBAU45436, and the DEGs on symbiotic plasmid pSF45436a were significantly depleted. This suggested that patterns of gene expression induced by WSHM are strain specific, except its universal promoting effect.

**FIG 3:**
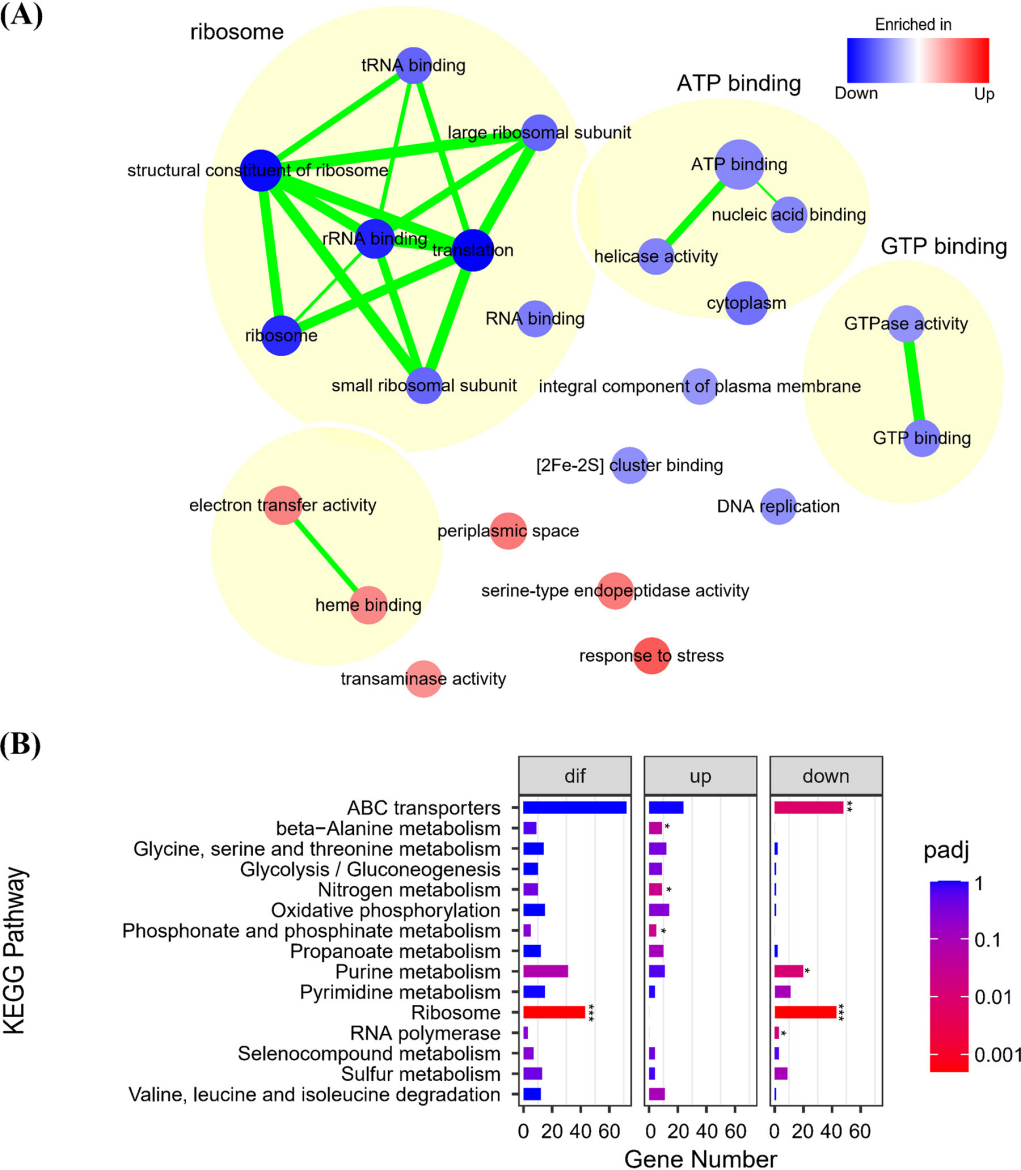
GO and KEGG enrichment analyses of DEGs. (A) GSEA delineates GO that is influenced by WSHM. Nodes represent enriched GO gene sets, whose size is proportional to the total number of genes, and red represents enrichment in WSHM treatment (i.e., upregulated in WSHM treatment), whereas blue represents enrichment in control (i.e., downregulated in WSHM treatment); color intensity is proportional to enrichment significance. Clusters of functionally related gene sets were manually circled and assigned a label. The number of overlapping genes between gene sets is represented as the thickness of the green line between nodes. (B) Histogram showing KEGG enrichment analysis of DEGs. The chart shows most enriched 15 pathways. (Fisher’s exact test with FDR correction: *, *P*_adj_ < 0.05; **, *P*_adj_ < 0.01; ***, *P*_adj_ < 0.001).

Functional enrichment analysis showed that upregulated genes were enriched in metabolic process and environmental response, while genetic information processes such as ribosome, ATP binding, GTP binding, and RNA binding were significantly inhibited (Fig. 3; Data set S1). Protein synthesis lies at the core of cell growth, and the ribosomal content exhibited positive linear correlations with the growth rate in the medium without restricted factors (35). However, it is difficult for bacteria to maintain the optimal state due to various limitations. While the cell global resources are constant, the increase in ribosomal proteins will inevitably lead to a decrease in the concentration of amino acids for other proteins. Therefore, to maximize cell growth, ribosome synthesis in cells must be precisely regulated according to the resource allocation model (36–39). In this study, the biological processes such as oxidative phosphorylation, nitrogen metabolism, and stress response were significantly upregulated, while ribosome synthesis were significantly downregulated upon WSHM treatment, suggesting that WSHM promoted the growth and survival of rhizobia by the tradeoff between the synthesis of ribosomes and metabolic proteins.

To validate the expression analysis of RNA-Seq data, changes in the expression of 19 genes from stress response, carbon/nitrogen metabolism, genetic information processing, and other processes were measured by quantitative real-time PCR (qRT-PCR) (Table S3). The good linear correlation between the RNA-Seq and qRT-PCR results demonstrated the reliability of RNA-Seq data (Fig. S4).

### Modulation of carbon and nitrogen metabolism.

**FIG 4:**
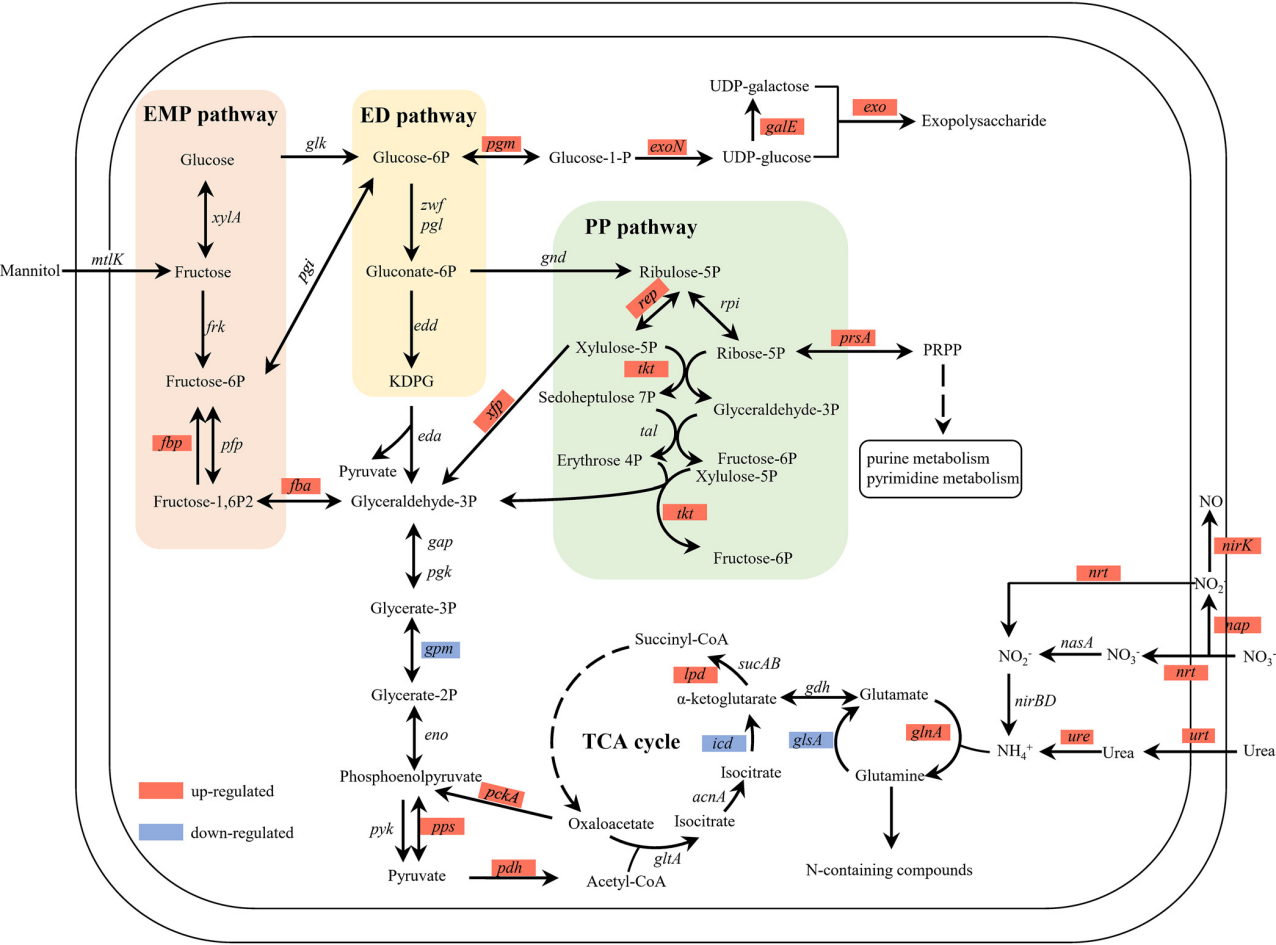
Effects of WSHM in carbon and nitrogen metabolism in *S. fredii* CCBAU45436. The metabolic network was reconstructed from the KEGG pathway. Each arrow shows the direction of the reaction. EMP pathway, ED pathway, and PP pathway are shown in salmon pink, pale yellow, and pale green box, respectively. Genes upregulated or downregulated by WSHM were highlighted in red or blue, respectively. EMP pathway, Embden-Meyerhof-Parnas pathway; ED pathway, Entner-Doudoroff pathway; PP pathway, pentose phosphate pathway; TCA, tricarboxylic acid cycle; KDPG, 2-keto-3-deoxy 6-phosphogluconate; PRPP, phosphoribosyl pyrophosphate.

**(i) Carbon metabolism.** Consistent with the increase of mannitol consumption in WSHM treatment, carbon metabolism of *S. fredii* CCBAU45436 was activated by WSHM (Fig. 4). Genes involved in the PP pathway, including *rpe* gene (log_2_fold change [FC] = 1.06, adjusted *P* value [*P*_adj_] < 0.01) encoding ribulose-phosphate 3-epimerase and *tkt* gene (log_2_FC = 1.78, *P*_adj_ < 0.001) encoding transketolase, were upregulated significantly. Meanwhile, *prsA* gene encoding ribose-phosphate pyrophosphokinase was significantly increased to 30.6 times greater than that of the control, which catalyzes the synthesis of phosphoribosyl pyrophosphate (PRPP) using the PP pathway intermediate ribose 5-phosphate as a substrate (Fig. 4; Table S4). PRPP is an important substrate for biosynthesis of nucleotides; the upregulation of *prsA* gene should be conducive to nucleotide synthesis and thus promote the growth of bacteria (40). Although the Entner-Doudoroff (ED) pathway is the main pathway of carbohydrate metabolism in rhizobia (41), it was found that a higher energy yield was obtained through PP pathway than through ED pathway when mannitol was used as a carbon source by Gluconobacter oxydans (42). It can be inferred that WSHM may increase the efficiency of energy production by activating PP pathway, which can also provide NADPH and precursor substances for biosynthesis of biological macromolecules (43). The product of glycolysis is converted into acetyl coenzyme A (acetyl-CoA) and oxidated in the tricarboxylic acid (TCA) cycle accompanied by the generation of NADH. Our data showed that the expression levels of *nuo* gene cluster encoding NADH dehydrogenase in WSHM treatment were 2.4- to 4.1-fold those in the control, and the expressions of *ccoO* and *ccoP* genes encoding *cbb_3_-*type cytochrome c oxidase, another important component of the cell electron transport chain, were upregulated by 21.2 and 17.5 times, respectively (Table S4). Oxidative phosphorylation driven by the electron transport chain is a primary way for bacteria to obtain the energy for growth and proliferation (44). Therefore, our results indicated that WSHM promoted the carbon metabolism, electron transport, and energy generation of *S. fredii* CCBAU45436.

**(ii) Nitrogen metabolism.** The results of transcriptome analysis also evidenced that WSHM regulated the nitrogen metabolism of *S. fredii* CCBAU45436. First, WSHM activated the uptake of nitrogen; the expression of *nrtABC* and *urtABCDE* genes encoding nitrate/nitrite and urea ABC transporter, respectively, were upregulated by 4 to 10 times in WSHM treatment (Fig. 4; Table S4). Correspondingly, the nitrogen assimilation was also significantly activated. The *nap* genes, encoding the periplasmic nitrate reductase participating in nitrate reduction, were significantly increased by 1.8 to 3.3 times (Fig. 4; Table S4), and they tend to be increased by the rate of reduction of nitrate to nitrite. The nitrite is reduced to NH_4_^+^, catalyzed by an assimilatory nitrite reductase encoded by *nirBD*, and then is converted to glutamine through glutamine synthetase (encoded by *glnA*, log_2_FC = 1.4, *P*_adj_ < 0.001). The glutamine further incorporates into nitrogen-containing compounds such as proteins and nucleic acids (45). On the other hand, nitrite was also reduced to NO by nitrite reductase encoded by *nirK* (log_2_FC = 3.8, *P*_adj_ < 0.001), and the expression of NnrS-encoding gene *nnrS* was significantly upregulated (log_2_FC = 3.56, *P*_adj_ < 0.001), protecting key metabolic pathways from NO inhibition (46). The gene expression of chaperone protein UreG, which is required for the assembly of urease enzyme complex, was significantly upregulated (*ureG*, log_2_FC = 1.06, *P*_adj_ < 0.001). This facilitated the hydrolysis of urea to ammonia and carbon dioxide, which may be one of the reasons why the pH of WSHM treatment is higher than that of the control. Thus, the results described above demonstrate that nitrogen metabolism was active in WSHM treatment, and the increase of nitrogen uptake and assimilation could help synthesis of proteins and nucleic acids and promote bacterial growth.

### Activation of stress defense response.

Bacteria frequently undergo many stressful conditions, such as nutrient deprivation, low pH (Fig. 2), reactive oxygen species (ROS), or accumulation of toxic by-products during the growth process. Improving the stress resistance of bacteria is of great benefit for their growth and application. The expression of stress response genes, including universal stress protein (USP), stress resistance chaperones, and antioxidant proteins, were significantly induced by WSHM. There were a total of nine copies of *usp* genes in *S. fredii* CCBAU45436 genome, seven of them with over 20-fold induction (Table 1). Envelope stress responses, including Cpx and σ^E^ systems involved in protection of envelope, were activated by WSHM, too. The *cpxP* gene, one of the targets of CpxRA two-component system, was most highly activated by WSHM and showed expression 132-fold higher compared to that in the control. The expression of protease-encoding genes *degS* and *degP*, which were involved in the proteolytic cascade of the σ^E^ response pathway, was significantly upregulated (3.6- to 11.9-fold) (Table 1). Also, some general stress proteins, such as chaperones (DnaK, GrpE, GroEL, and GroES) and heat shock proteins (Hsp), were 2.2- to 38.2-fold more abundant in WSHM treatment. In addition, the expression level of antioxidant defense genes, including the two best characterized redox-sensing proteins OxyR and SoxR, encoded by genes *oxyR* and *soxR*, and their regulon *katG*, was significantly increased by 4.4-, 2.4-, and 2.2-fold, respectively.

**TABLE 1:** The activation of stress response genes by WSHM

Stress response gene type	Gene ID	Gene name	Log_2_FC	*P*_adj_	Gene product
Universal stress protein	AB395_RS03245	*usp*	4.73	4.10E−106	Universal stress protein
	AB395_RS04425	*uspA*	5.68	4.40E−175	Universal stress protein UspA
	AB395_RS32640	*uspA*	5.05	1.32E−93	Universal stress protein UspA
	AB395_RS32670	*uspA*	4.3	2.13E−60	Universal stress protein UspA
	AB395_RS32750	*uspA*	5.64	1.50E−101	Universal stress protein UspA
	AB395_RS32755	*uspA*	5.62	2.40E−143	Universal stress protein UspA
	AB395_RS32375	*uspA*	4.81	2.25E−49	Universal stress protein UspA
Chaperone	AB395_RS32850	*cpxP*	7.04	7.20E−169	Spy/CpxP family protein refolding chaperone
	AB395_RS32695	*degS*	3.58	4.22E−49	Outer membrane stress sensor protease DegS
	AB395_RS32460	*degP*	5.12	1.45E−21	HtrA protease/chaperone/serine protease
	AB395_RS27470	*degP*	3.54	9.82E−78	Do family serine endopeptidase
	AB395_RS03445	*degP*	1.86	4.1E−15	Do family serine endopeptidase
	AB395_RS32645	*groEL*	5.26	2.44E−80	Heat shock protein 60 family chaperone GroEL
	AB395_RS31465	*groES*	1.26	2.68E−07	Heat shock protein 60 family cochaperone GroES
	AB395_RS04275	*groEL*	1.11	5.13E−07	Heat shock protein 60 family chaperone GroEL
	AB395_RS32665	*hsp*	4.45	2.04E−92	Molecular chaperone (small heat shock protein)
	AB395_RS06015	*clpA*	1.01	4.31E−05	ATP-dependent Clp protease ClpA
	AB395_RS13640	*clpB*	1.61	4.41E−13	ClpB protein
Antioxidant protein	AB395_RS02005	*oxyR*	2.13	6.67E-31	Hydrogen peroxide-inducible genes activator
	AB395_RS08275	*soxR*	1.25	1.18E−07	Redox-sensitive transcriptional activator SoxR
	AB395_RS25255	*katG*	1.15	2.69E−08	Catalase/peroxidase HPI

The expression products of the genes induced by WSHM play important roles in bacterial defense against stress. The USP, which is widely distributed in both prokaryotes and eukaryotes, is reported to be stimulated under hostile environments to help cells to survive (47). CpxP and DegP play an important role in degradation of damaged protein or remodeling of misfolded protein. The growth of E. coli mutants *cpxP* or *degP* is inhibited when the misfolded PapE is synthesized (48), and the deletion of *degP* gene in E. coli resulted in decreases of viability and cellular resistance to extreme acid stress of the mutant (49). Chaperones can effectively protect proteins against misfolding when cells undergo stress. It was found that most heat-tolerant rhizobia showed induction of chaperone genes higher than that of heat-sensitive strains under temperature stress, and overexpression of *groESL* genes in E. coli enhanced the tolerance of strains to heat stress (50, 51). In fact, fast growth is a stressor of the cell, causing proteins to be expressed rapidly and aggregate easily. However, the protein aggregation could be prevented by overexpression of chaperones to protect proteins against misfolding and maintain a high level of protein expression simultaneously (52, 53). Excessive ROS are detrimental, causing oxidative damage to DNA and lipids and, ultimately, cell death (54). Transcription factors OxyR and SoxR play a key role in protecting bacteria against oxidative stress (55, 56). OxyR is a primary regulator that modulates H_2_O_2_ scavenging activity and regulates the expression of numerous oxidative defense-related genes, such as *katG*. It has been found that deletions of *oxyR* or *katG* in Azorhizobium caulinodans lead to significantly reduced ROS resistance and symbiotic nitrogen fixation efficiency (57). SoxR and its regulons confer mainly resistance to superoxide, and one of the regulons, superoxide dismutase (SOD), represents a first line of defense against reactive oxygen (58). The data of enzyme assay showed that SOD activities of *S. fredii* CCBAU45436 growth in WSHM treatment were significantly (2.6- and 2.1-fold) greater than those in the control at 24 h and 48 h, respectively (Fig. S5). Consistent with the significant upregulation of *exo* gene clusters (Fig. 4; Table S4), we detected that the exopolysaccharides (EPS) content in culture was significantly increased by 1.12-fold in WSHM treatment (Fig. S6). EPS have been considered to play important roles in protecting rhizobia against stress and the symbiosis process (59). These results demonstrated that WSHM promoted rhizobial growth and survival by activating cellular stress responses such as envelope stress response, molecular chaperone, and oxidative stress response.

### Gene *rsh* is a hub in WSHM function.

To identify hub genes involved in rhizobial response to WSHM, a protein-protein interaction (PPI) network of the DEGs was constructed by way of the STRING database. There are 62 proteins with node degree greater than 50, whose UniProt keywords were “ribosomal protein,” “protein biosynthesis,” “RNA polymerase,” “GMP biosynthesis,” and others, respectively (Fig. S7; Data set S2). Among the rest of the nine proteins, the RSH caught our attention because it was annotated as a bifunctional (p)ppGpp synthetase/hydrolase. Then, we ran a sequence of RSH against the Pfam database and found that it was composed of four domains: (p)ppGpp hydrolase domain (HD), RelA/SpoT (p)ppGpp synthase domain (RelA_SpoT), and two regulatory domains, TGS domain and ACT domain, showing the typical domain organization characteristics of RelA-SpoT homologs (Fig. S8). The whole-genome alignment analysis showed that there was not another homologous protein in the genome, which means that the (p)ppGpp level of *S. fredii* CCBAU45436 is controlled only by this RSH protein.

**FIG 5:**
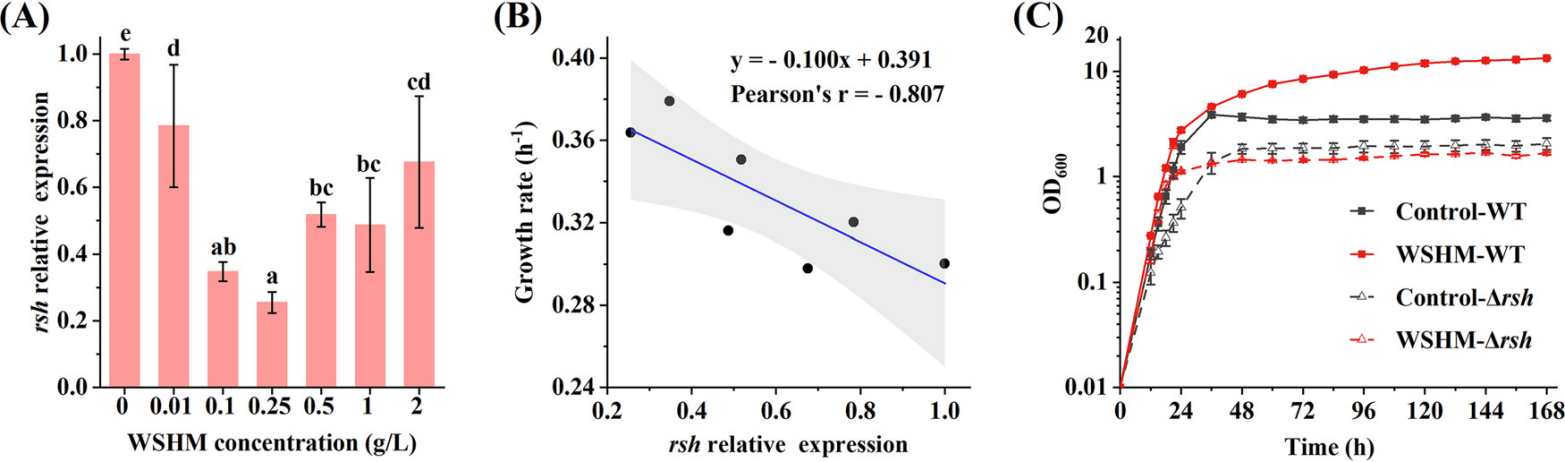
Gene *rsh* is a hub in WSHM function. (A) Relative expression levels of *rsh* gene in YM broth supplied with different concentrations of WSHM. Different letters above the error bar indicate a significant difference between means based on Duncan’s test (α = 0.05). (B) The linear correlation between growth rate and *rsh* gene expression. The gray region indicates 95% confidence intervals. (C) Effects of WSHM on the growth of *S. fredii* CCBAU45436 WT and Δ*rsh* in YM broth. The data are presented as the means ± standard deviations from triplicate experiments (A and B) and from quadruplicate experiments for WT and four independent clones for Δ*rsh* (C).

Transcriptome analysis showed that the *rsh* gene (log_2_FC = −1.1, *P*_adj_ < 0.001) was significantly underexpressed in WSHM treatment. To verify the regulation of WSHM on rhizobial metabolism, we tested the effects of different concentrations of WSHM on the expression of *rsh* gene. The expression level of *rsh* gene was the highest in the control and was dramatically decreased in treatments with different concentrations of WSHM. The lowest expression level of *rsh* gene occurred in the range of 0.1 to 1 g/liter WSHM and was raised in either lower or higher concentration (Fig. 5A). The pattern of *rsh* gene expression in different concentrations of WSHM seems to be exactly inverse to promoting effects (Fig. 1A), and general linear regression analysis showed that the growth rate decreased with increasing expression level of *rsh* gene (Pearson’s *r* = −0.807, *P < *0.05) (Fig. 5B). To determine the role of RSH in the effects of WSHM on rhizobial growth, an in-frame deletion mutant of *rsh* gene (Δ*rsh*) was constructed for *S. fredii* CCBAU45436 and the effect of WSHM on Δ*rsh* was tested. The results showed that WSHM did not increase the cell density of Δ*rsh* (Fig. 5C), indicated that RSH is one of the upstream proteins in response to WSHM, and showed that WSHM indeed promoted the growth of rhizobia by regulating the metabolism and even global resource allocation. However, WSHM still increased the exponential growth rate of Δ*rsh*, suggesting that uncharacterized pathways independent of RSH might be involved in WSHM-induced growth in “optimal” conditions. The Δ*rsh* mutant showed slower growth than that of the wild type (WT) and never reached the same saturation level, meaning that the presence of RSH plays an important role in growth of the tested bacterium.

### Increases of root colonization and viability in soil of *S. fredii* by WSHM.

**FIG 6:**
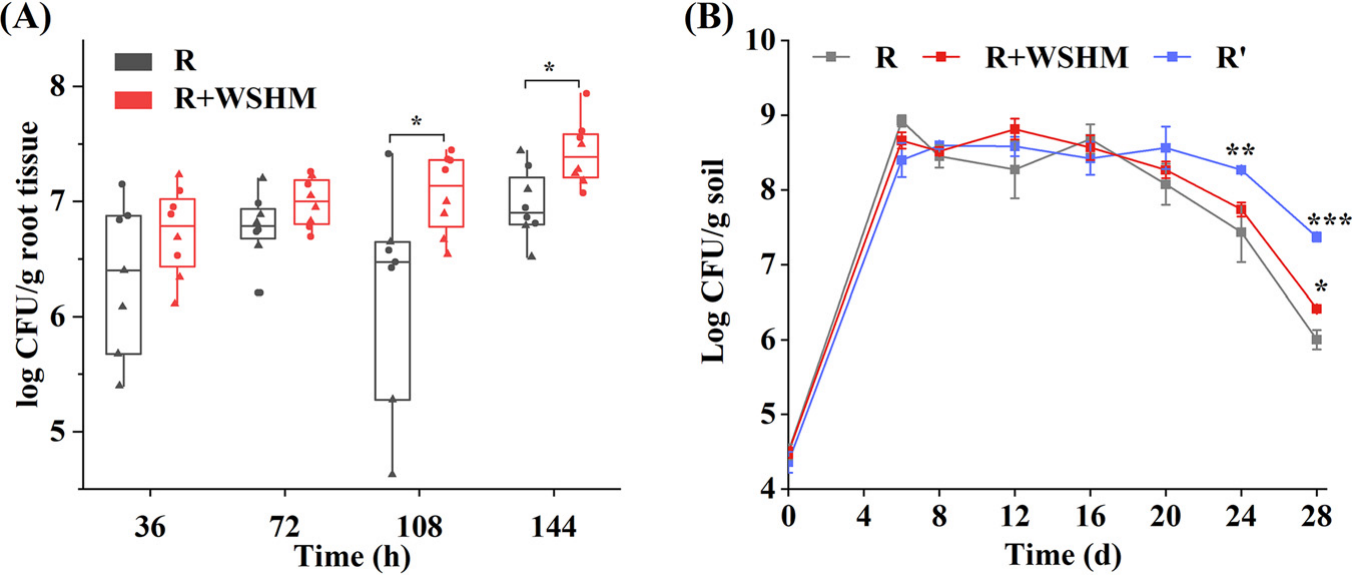
Effects of WSHM on root colonization (A) and the viability in soil (B) of *S. fredii* CCBAU45436. R, cells cultured in YM broth; R+WSHM, cells cultured in YM broth and coinoculation with WSHM; R’, cells cultured in YM broth supplied with WSHM. The shapes represent two independent experiments in panel A. Significant difference between the mean of control and the corresponding value of WSHM treatment is indicated. Wilcoxon rank-sum test (A) or *t* test (B): *, *P* < 0.05; **, *P < *0.01; ***, *P < *0.001.

Root colonization and survival in soil of rhizobia are prerequisites for the mutualism between rhizobia and their hosts and are important standards for the quality control of rhizobia inoculant (60, 61). In the present study, the effect of WSHM on the colonization of *S. fredii* CCBAU45436 on soybean roots was assayed. The results showed that WSHM promoted the colonization of *S. fredii* CCBAU45436 in rhizoplane; the rhizobial number in WSHM treatment (7.22 × 10^6^ CFU/g root tissue) was 53.3% higher than that of the control (4.71 × 10^6^ CFU/g root tissue) 36 h after inoculation and increased significantly 1.6 and 1.8 times at 108 h and 144 h, respectively (Fig. 6A).

We further studied the effect of WSHM on the viability of *S. fredii* CCBAU45436 in soil. It has been reported that stress response proteins induced in sublethal stress condition were likely to protect bacteria from subsequent stress (62, 63); for example, Draghi et al. (63) showed that acid-adapted rhizobia (ATR+) presented a clear advantage in survival when exposed to lethal low pH. So, we hypothesized that the stress defenses activated by WSHM in free-living cultures might aid the rhizobia survival in soil. To test this hypothesis, in addition to single inoculation of *S. fredii* CCBAU45436 cultured in YM broth (R) or its coinoculation with WSHM (R plus WSHM), cells cultured in YM broth supplied with WSHM (R’) were also inoculated in the soil. The counting of viable cells showed that the number of viable bacteria initially increased in the first 6 days, followed by stable state above 10^8^ CFU/g soil until the 16th day in all three treatments. Then, the viable cells in group R showed a more rapid decline than that in group R plus WSHM and R’; on the 28th day, the group R’ had the highest number of viable bacteria (2.36 × 10^7^ CFU/g soil), followed by the groups R plus WSHM (2.60 × 10^6^ CFU/g soil) and R (1.03 × 10^6^ CFU/g soil) (Fig. 6B). The above results demonstrated that WSHM treatments in the culture stage (R’) or in the postculture stage (R plus WSHM) improved the survival of rhizobia in soil with some unclear mechanism.

A large number of pre- and cross-adaptation experiments of microbes have shown that the stress-related proteins induced under sublethal stresses may trigger the formation of cross-protection to defense against subsequent stress (62, 64). Previous studies on plants found that a low concentration of humic acid promoted plant growth by causing mild stress on water absorption of roots (65); WSHM may cause slight stress on *S. fredii* and triggered the stress response to aid rhizobial growth and survival in a later unfavorable environment.

### Conclusion.

WSHM significantly promoted the growth and survival of *S. fredii* CCBAU45436 in YM broth in a bell-shaped, dose-dependent manner, along with improvement of carbon source utilization efficiency and slow-down of medium acidification. Comparative transcriptome analysis showed that WSHM modulated the metabolism and triggered cellular stress defense but inhibited genetic information processing. The key hub gene *rsh* was found by a PPI network analysis and validated by qRT-PCR and in-frame deletion mutant, suggesting that the WSHM functioned by regulating the metabolism and even global resource allocation. In addition, WSHM were beneficial to root colonization and viability in soil of *S. fredii* CCBAU45436. Conclusively, this study shed new insights into the effects of WSHM on bacterial adaptations and demonstrated that it can be a unique high-efficiency inoculant additive. Furthermore, the mechanism of WSHM promoting bacterial growth may provide candidate genes for improving environmental adaptability and application potential of bacteria through genetic engineering.

## MATERIALS AND METHODS

### WSHM, bacterial strains, plasmids, and growth conditions.

WSHM were extracted from lignite collected from the Huolingele Minerals Administration in Inner Mongolia, China as previously described, which contains 49.7% C, 3.7% H, 2.5% N, and 43.6% O, respectively (66). By tetramethyl ammonium hydroxide (TMAH)-py-GC/MS analysis, 68 compounds belonging to four classes (aromatics, aliphatic, nitrogen compounds, and other compounds) were detected in the WSHM (21).

Bacterial strains and plasmids used in this study are listed in Table S5. Among them, Sinorhizobium fredii strain CCBAU45436 was provided by Rhizobium Research Center, China Agricultural University, Beijing, China. This strain was an effective symbiont of soybean isolated from Henan Province, China (67). *S. fredii* strain and its mutants were grown at 28°C in tryptone-yeast extract (TY) medium (68) or YM broth (21). E. coli strains were grown at 37°C in Luria-Bertani (LB) medium. The final concentrations of antibiotics used were 50 μg/ml for kanamycin (Km), 30 μg/ml for gentamicin (Gen), and 30 μg/ml for nalidixic acid (NA) when required.

### Construction of *rsh* deletion mutant.

Primers used for mutant construction are listed in Table S5. To construct the in-frame deletion mutant of *rsh*, a 616-bp upstream region and a 568-bp downstream region flanking the *rsh* coding sequence were amplified and cloned into the suicide vector pJQ200SK by using the seamless assembly and cloning kit (GenStar). The resultant pJQ200SK derivative with correct cloned sequences, verified by Sanger sequencing, was then introduced from E. coli DH5α into *S. fredii* CCBAU45436 with helper plasmid pRK2013 by triparental conjugation. Single-crossover clones resistant to Gen were selected for subsequent cultivation in liquid TY medium for 24 h. The resulting liquid culture was subjected to double-crossover screening on TY plate containing 5% sucrose (wt/vol) and NA. The mutants were confirmed by colony PCR using primers *rsh*-WF/*rsh*-WR and subsequent Sanger sequencing.

### Rhizobial growth monitoring.

A fresh colony of *S. fredii* CCBAU45436 WT was inoculated into 4 ml of YM broth and incubated at 28°C with 200 rpm shaking for several hours up to the exponential growth phase. Then, the culture was inoculated at an initial OD_600_ of 0.01 into 50 ml of YM broth supplied with WSHM at the final concentrations of 0, 0.01, 0.1, 0.25, 0.5, 1, and 2 g/liter, respectively. The inoculated flasks were incubated as mentioned above, and OD_600_ was monitored every 3 h in exponential growth phase for growth and then every 12 h or 24 h for survival. The cultures of control (0 g/liter WSHM) and treatment with 0.5 g/liter WSHM were sampled, serially diluted, and plated on TY agar every 24 h for counting of the CFU after incubation.

To test whether WSHM were used directly as a carbon or nitrogen source, YM broth supplied with 0.629 g/liter mannitol alone, 0.048 g/liter NH_4_Cl alone, or both together, which correspond to the same amount of carbon and nitrogen in 0.5 g/liter WSHM, was inoculated with culture of *S. fredii* CCBAU45436 as mentioned above. Also, to test whether the effect of WSHM relies on its modification of pH in the culture, YM broth supplied with 0.5 g/liter WSHM, 20 mM MOPS (pH adjusted to 6.8 by 1 M NaOH), or both was also inoculated as mentioned above. The incubation conditions were the same as mentioned above, and OD_600_ and pH of the cultures were monitored every 24 h. To determine the role of RSH in the WSHM-induced growth promotion, precultures of *S. fredii* CCBAU45436 WT and Δ*rsh* were growth in 50 ml YM broth or YM broth supplied with 0.5 g/liter WSHM. The incubation conditions were the same as mentioned above, and OD_600_ were monitored.

### Mannitol consumption detection.

Aliquots of 1 ml bacterial culture in control or treatment of WSHM (0.5 g/liter) were sampled every 24 h for 10 days and then every 3 days for two times. Samples were centrifuged at 12,000 × *g* for 15 min, and the concentrations of mannitol in the supernatant were determined by Essentia LC-15C high-performance liquid chromatography (HPLC) (Shimadzu, Kyoto, Japan) using a Remex ROA-organic acid H^+^ (8%) column (Phenomenex, Los Angeles, CA, USA) and a RID-10A refractive index detector (Shimadzu, Kyoto, Japan). In this analysis, pH and OD_600_ were also determined simultaneously. Three independent experiments were performed.

### RNA extraction and sequencing.

*S. fredii* CCBAU45436 was cultured in control or WSHM treatment (0.5 g/liter) as mentioned above for analysis of transcriptome. Samples were harvested at 24 h by centrifugation, and total RNA was extracted using the Eastep super total RNA extraction kit (Promega, Shanghai, China) according to the manufacturer’s instructions. Quality and integrity of the extracts were determined using a NanoDrop spectrophotometer (Thermo Scientific) and a Bioanalyzer 2100 system (Agilent). Then, the library construction, sequencing, data filtering, and mapping were performed by commercial service in Shanghai Personal Biotechnology Co., Ltd., China. The sequencing library was sequenced with Illumina HiSeq X 10 platform, and three independent experiments were performed.

### RNA-Seq data analysis.

Bowtie2 was used to map clean reads to the *S. fredii* CCBAU45436 genome (PRJNA285929). The level of gene expression was estimated by the expected number of transcripts per million reads (TPM), and differentially expressed genes (DEGs; |log_2_ FC| ≥ 1 and false-discovery rate [FDR] < 0.05) were identified by DESeq2. Gene Set Enrichment Analysis (GSEA) was used to find enriched Gene Ontology (GO) gene sets in DEGs by GSEA software (69) performed with an algorithm with 1,000 permutations, minimum term size of 5, and maximum term size of 500. Only gene sets passing conservative significance thresholds (*P < *0.05 and FDR < 0.25) were selected for display in the Enrichment Map of Cytoscape (70). The Kyoto Encyclopedia of Genes and Genomes (KEGG) enrichment analysis was conducted by using the “clusterProfiler” R package and Fisher’s exact test, while *P* values were adjusted with the BH method. The PPI network was generated by STRING version 11.0 (https://version-11-0.string-db.org/) (71) and visualized with Cytoscape. RSH protein sequence was run against the Pfam database by HMMER online (http://www.ebi.ac.uk/Tools/hmmer/) to identify homologies and domain architecture.

### qRT-PCR.

*S. fredii* CCBAU45436 was grown in YM broth supplied with WSHM at concentrations of 0, 0.01, 0.1, 0.25, 0.5, 1, and 2 g/liter, respectively. The cells were collected at 24 h and total RNA was extracted as described above. Then, cDNA was synthesized using GoScript reverse transcriptase (Promega, USA). qRT-PCR was performed according to the protocol of the 2× RealStar green power mixture (with ROX II) (Genestar, Beijing, China). Gene-specific primers used in qRT-PCR are listed in Table S3. To validate the results of RNA-Seq, the expressions of 19 genes with different functional categories of the control (0 g/liter WSHM) and 0.5 g/liter WSHM treatment were measured by qRT-PCR. Gene expression was standardized to that of the 16S rRNA gene for all samples. The expressions of *rsh* gene in treatments of 0.01, 0.1, 0.25, 0.5, 1, and 2 g/liter of WSHM were also tested, standardized to the 16S rRNA gene, and further normalized to the control. All tests were performed in three biological and three technical replicates.

### Measurement of SOD activity and EPS.

Aliquots of 5 ml CCBAU45436 cultured in control or treatment of WSHM (0.5 g/liter) were collected by centrifugation at 24 h and 48 h. EPS content at 24 h was precipitated from the culture supernatant at 4°C for 30 min using 4 volumes of 95% ethanol, and then the samples were centrifuged and the obtained EPS was dried and assayed by the anthrone-sulfuric acid method (72). For measurement of SOD activity, the cell pellet was washed twice with saline solution and adjusted to an OD_600_ of 1. Then, 3 ml of this cell suspension was centrifuged and resuspended in 1 ml saline solution for cell lysis by sonication on ice for 10 min. The lysate was centrifuged at 6,000 rpm at 4°C for 10 min, and the supernatant was used for measurement of SOD activity based on the manufacturer’s instructions of superoxide dismutase assay kit (WST-1 method) (Jiangcheng, Nanjing, China). SOD activities were further normalized by the total protein concentration of the crude extracts.

### Root colonization assays.

Inoculum was prepared as described by diCenzo et al. (73) with modification. Briefly, *S. fredii* CCBAU45436 was cultured in 50 ml of YM broth as mentioned above for 24 h, cells were harvested by centrifugation and washed twice with phosphate-buffered saline (PBS), and the cell pellet was resuspended to an OD_600_ of 0.02 (approximately 2 × 10^7^ CFU/ml) with PBS (R) or PBS supplied with 0.5 g/liter of WSHM (R plus WSHM) according to the treatment. Seeds of Glycine max cv. Xudou18 were surface sterilized and allowed to germinate as described earlier (32). Then, four seedlings were transferred to each square petri dish (13 by 13 cm) containing 0.8% agar and covered by a piece of sterilized filter paper (11 by 11 cm). After that, the roots of seedlings were covered by another, smaller sterilized filter paper (11 cm by 8 cm) to maintain darkness of the root. Aliquots of 5 ml inoculum were added to the plate according to treatment, and plates were sealed with Parafilm M (Parafilm), returned to the growth chamber, and grown vertically with random shuffling. Bacterial cells colonized on soybean rhizoplane were recovered every 36 h by washing the root with 60 ml sterilized PBS 2 times and then treated with ultrasound for 30 s at 80 Hz in a tube with 40 ml PBS. The ultrasonic suspension was diluted and plated on TY plates with NA antibiotics. The CFU numbers were determined per gram of root fresh weight. Four replicates in two independent experiments were performed.

### Survival assay of *S. fredii* in soil.

As the *S. fredii* populations are widely distributed and dominant in alkaline-saline soil (74), a soil with pH 7.60 was collected from a soybean field in Jining of Shandong Shofine Seed Technology Co. Ltd. The soil physiochemical properties have been determined previously by Yang et al. (75). The sampled soil was passed through a 2-mm sieve. Microcosms were constructed with 10 g of the sieved soil in 50 ml serum bottles and autoclaved (121°C, 30 min) three times with intervals of 24 h. Inoculum was prepared as mentioned above, except the cell pellet was resuspended to an OD_600_ of 0.001 (approximately 10^6^ CFU/ml). Furthermore, *S. fredii* CCBAU45436 cultured in YM supplied with 0.5 g/liter WSHM was also resuspended in PBS as R’. For each microcosm, 1 ml prepared cell dilution was mixed thoroughly with soil, and the microcosms were incubated at room temperature (25 ± 2°C) in the dark. To determine the number of viable cells, 0.05 g soil was sampled on the 6th and 8th days for the first two times and then every 4 days from each microcosm and serially diluted with PBS to 10^−6^. Then, an aliquot of 0.1 ml of the dilution 10^−4^ to 10^−6^ was plated in duplicate on TY agar, the plates were incubated at 28°C for 3 to 4 days, and colonies were counted for calculating viable cells per gram of soil.

### Statistics analysis.

Results were showed as mean ± standard deviation (SD) using Origin2021, and statistical analyses were carried out using the SPSS 23.0 software.

### Data availability.

The RNA-Seq data have been submitted to the SRA databases under accession number PRJNA682353.
