# Supplementary material for: Water-Soluble Humic Materials Modulating Metabolism and Triggering Stress Defense in Sinorhizobium fredii
Source: Microbiol Spectr. 2021 Aug 25;9(1):10.1128/spectrum.00293-21. doi: 10.1128/spectrum.00293-21 (PMC8552645; doi:10.1128/spectrum.00293-21)
Supplement: SUPPLEMENTAL FILE 2 — Supplemental material. Download SPECTRUM00293-21_Supp_1_seq9.pdf, PDF file, 0.7 MB [file spectrum00293-21_supp_1_seq9.pdf]

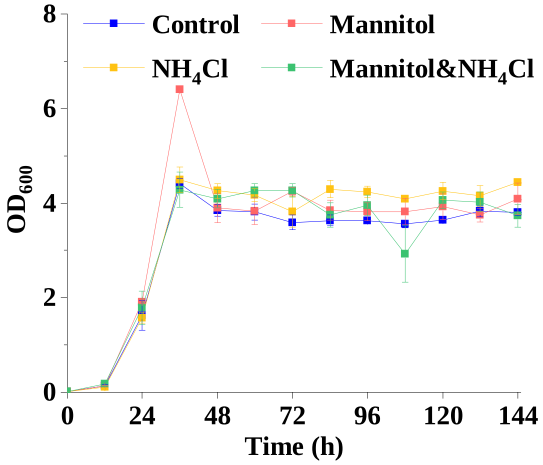

Fig. S1. Effects of external addition of mannitol or NH_4Cl with an equivalent amount of carbon or nitrogen in 0.5 g/L WSHM on the growth of *S. fredii* CCBAU45436. The data are presented as the means ± standard deviations from triplicate experiments.

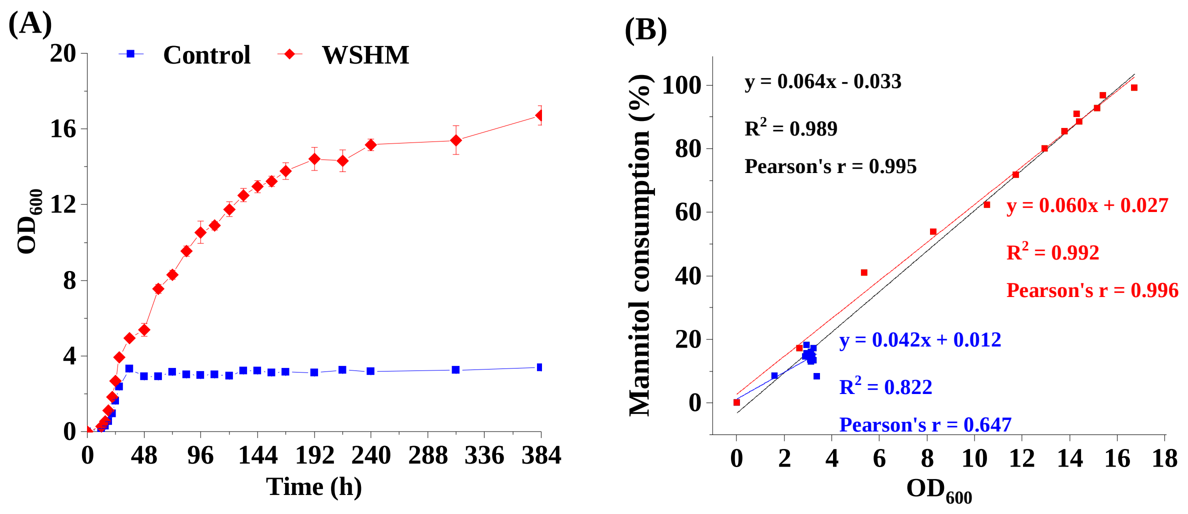

Fig. S2. Effects of WSHM on the growth and carbon source utilization efficiency of *S. fredii* CCBAU45436. (A) Effects of WSHM on the growth. (B) Correlations between mannitol consumption and OD_{600}. Blue, red and black represent samples collected from YM broth or YM broth supplied with 0.5 g/L WSHM and all, respectively. The linear model between mannitol consumption and OD_{600} were shown.

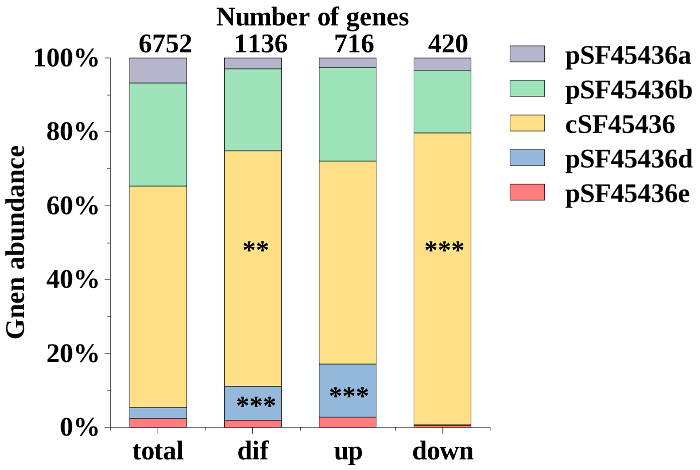

Fig. S3. Distribution of genes among replicons; total: all genes of *S. fredii* CCBAU45436, dif: differentially expressed genes, up: up-regulated genes, down: down-regulated genes; (Fisher's exact test with FDR correction, **, padj < 0.01; ***, padj < 0.001, only significantly enriched gene cluster were marked).

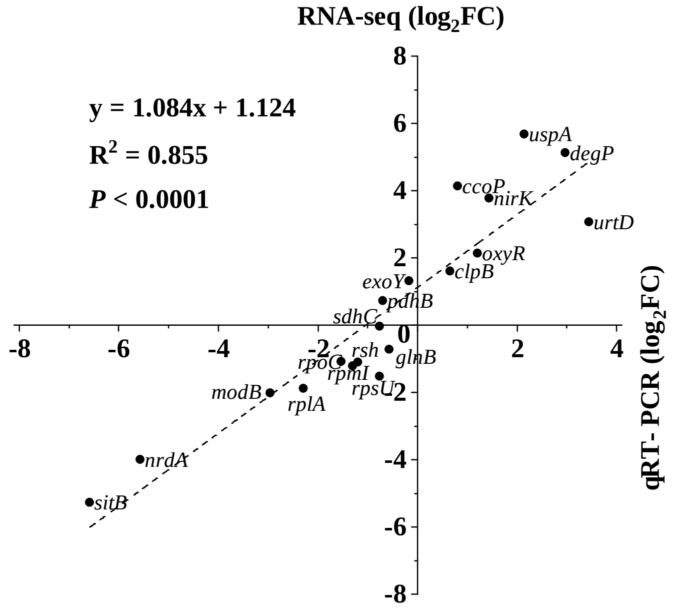

Fig. S4. Comparisons of fold change values (log_2FC) of selected genes from the different functional categories measured by qRT-PCR and RNA-Seq. The R^2 indicates the square of Pearson's correlation coefficient r.

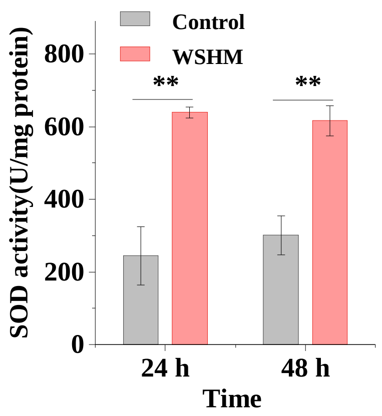

Fig. S5. Effects of WSHM on superoxide dismutase activity of *S. fredii* CCBAU45436 at 24 h and 48 h; (t-test, **, $P < 0.01$). The data are presented as the means ± standard deviations from triplicate experiments.

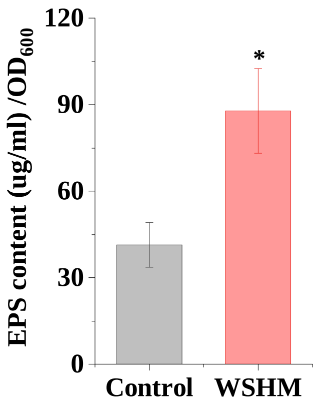

Fig. S6. Effects of WSHM on exopolysaccharides (EPS) production of *S. fredii* CCBAU45436 at 24 h; (t-test, *, $P < 0.05$). The data are presented as the means ± standard deviations from triplicate experiments.

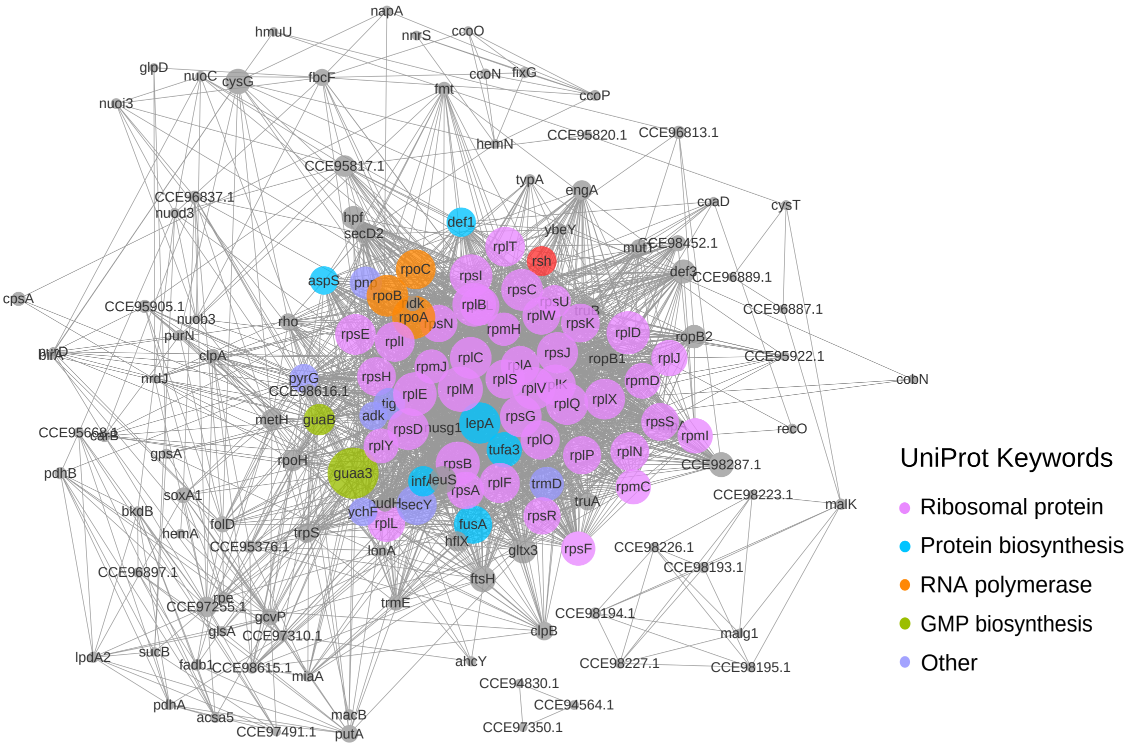

Fig. S7. STRING protein-protein interaction network for the significant differentially expressed genes, only the node with degree > 10 were shown. The size of the node is proportional to the degree, and nodes with degree ≥ 50 are colored according to the UniPort Keywords of protein expect *rsh* gene was shown in red.

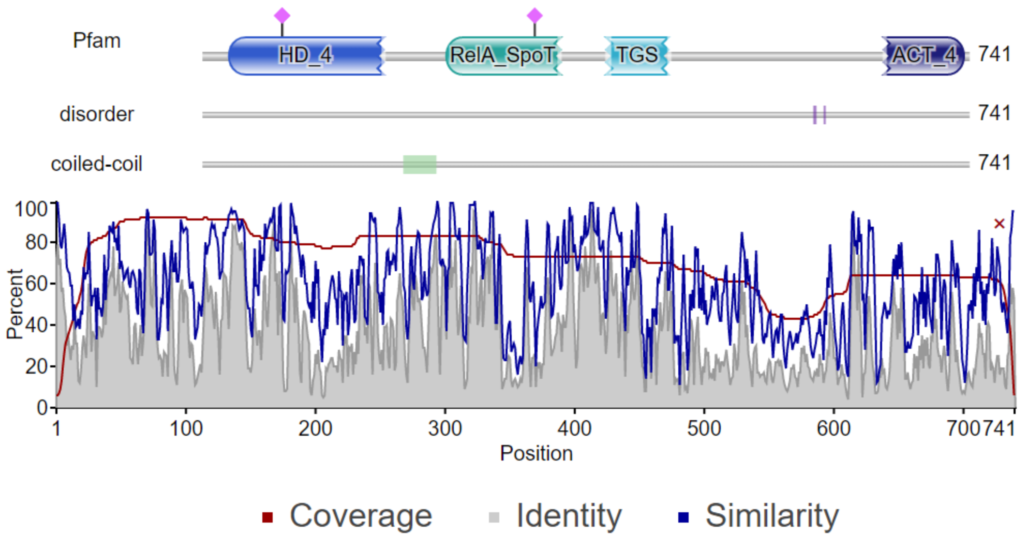

Fig. S8. Sequence matches and features of the *S. fredii* CCBAU45436 RSH protein against the Pfam database. HD: hydrolase domain; RelA-SpoT: predicted RelA-SpoT ppGpp synthase domain; TGS: Threonyl-tRNA synthetase, GTPase and SpoT; ACT: Aspartokinase, Chorismate mutase and TyrR.

Table S1. Data quality of RNA-Seq datasets and genomic alignment results of sequencing reads

Sample name	Raw reads	Clean reads	Q20(%)	Q30(%)	Total mapped	Uniquely mapped
Control1	19532312	19005078	98.39	94.91	18790049<br>98.87%	18122679<br>95.36%
Control2	17384566	17185396	98.82	96.08	16947252<br>98.61	16269213<br>94.67%
Control3	14259850	14096340	98.81	96.04	13922282<br>98.77%	13394195<br>95.02%
WSHM1	14957640	14779534	98.82	96.08	14585780<br>98.69%	14225887<br>96.25%
WSHM2	20254278	20034488	98.82	96.06	19777881<br>98.72%	19282765<br>96.25%
WSHM3	14233518	13882542	98.5	95.22	13733661<br>98.93%	13406660<br>96.57%

Table S2. Distribution of genes among replicons, p-adjust: Fisher's exact test with FDR correction. The p-adjust of significantly enriched gene cluster were bold.

replicons	total gene	DEG	% of dif	p-adjust	up-regulated	% of up	p-adjust	down-regulated	% of down	p-adjust
pSF45436a	452	33	2.90%	3.6E-09	19	2.65%	1.7E-06	14	3.33%	0.00405
pSF45436b	1888	252	22.18%	2.3E-06	181	25.28%	0.14	71	16.90%	1.5 E-07
cSF45436	4051	725	63.82%	**0.00484**	393	54.89%	0.00902	332	79.05%	**6.7E-17**
pSF45436d	203	104	9.15%	**1.1E-29**	103	14.39%	**6.5E-48**	1	0.24%	7.6E-05
pSF45436e	158	22	1.94%	0.38886	20	2.79%	0.36041	2	0.48%	0.00416
sum	6752	1136	100.00%		716	100.00%		420	100.00%	

Table S3. Genes selected from different functional categories and gene-specific primers for qRT-PCR analysis

Gene ID	Gene name	Primer (5'-3')	Functional categories	Gene production
AB395_RS18320	16S	GTGATAAGCCGAGAGGAAGGT<br>CCACTGTCACCACCATTGTAG		16S rRNA gene (the reference)
AB395_RS04425	*uspA*	AGGAGGTTCTCAATCAGCAT<br>ATCATCGCCTTCGTCACA	stress response genes	Universal stress protein UspA and related nucleotide-binding proteins
AB395_RS32460	*degP*	TCGCTTACGGAGAATACA<br>GTTCACGACAAGCCTTAG		Trypsin-like serine proteases
AB395_RS02005	*oxyR*	TCTGGTGCCGCAACTCATTC<br>CCTGTCGGTCAGCGATTCC		Hydrogen peroxide-inducible genes activator
AB395_RS13640	*clpB*	AATACGAGCAGCACCACAAGG<br>CCGTGATGTAGCGATTGGATAGG		ATP-dependent chaperone ClpB
AB395_RS23120	*exoY*	CCGCAACGATGTGTCCTA<br>CTTGAAGACGATGACGAGGT	carbon metabolism	Exopolysaccharide biosynthesis polyprenyl glycosyl phosphotransferase
AB395_RS05810	*pdhB*	GCTCTCCAAGTGGCTGAA<br>CTCCATCGTCGCCTTGTC		pyruvate dehydrogenase complex E1 component subunit beta
AB395_RS16895	*sdhC*	CTCTCGCCGCATCTTCAA<br>CACCAGGCAACCAGGATC		succinate dehydrogenase, cytochrome b556 subunit
AB395_RS08875	*ccoP*	CCTCGCTGGTGGGTCTAC<br>CTTCGTCGCATCCGTCAAC		Cbb3-type cytochrome c oxidase subunit
AB395_RS04540	*nirK*	GGCGAGCAGGACTTCTATGT<br>TTCCAGCGTATCGGCGTAG	nitrogen metabolism	Copper-containing nitrite reductase
AB395_RS21215	*urtD*	AACTCGCTCTCCTTCATC<br>GTCCATCATCGTCGTCTT		urea ABC transporter ATP-binding protein UrtD
AB395_RS07010	*glnB*	CGGACGAGAATGCGGAAG<br>TGCGGATTCGGATCACTT		nitrogen regulatory protein P-II
AB395_RS05290	*rpoC*	AGATTCGTGGTCGCTACAAG<br>GGTTCTTCGGCAGGAGTT	genetic information processing	DNA-directed RNA polymerase subunit beta'
AB395_RS19775	*rpmI*	AGCGTTCCAACAAGTTCATTC<br>CGTTCGGCAGGTAGTTCT		50S ribosomal protein L35
AB395_RS15575	*rpsU*	GCAGGTACTTGTCCGCGATAAC<br>TGCGACGGCTTTTCATAGAAGTC		30S ribosomal protein S21
AB395_RS05270	*rplA*	GCATTGATCCGGTGAAGG<br>GAGATTCATCGCCACTTCG		50S ribosomal protein L1
AB395_RS29160	*nrdA*	TCGCTGACGCTGTTCTTC<br>ATGGTCTTGATGCCCTTCTT		class 1b ribonucleoside-diphosphate reductase subunit alpha
AB395_RS03645	*rsh*	GAGGAGGAACTGAGCGAACTG<br>GCGGAAGACGGAATAGGGTTT	others	bifunctional (p)ppGpp synthetase/hydrolase

AB395_RS15960	*modB*	GTGCTGCTCATCCTGTTC<br>AGCGGAAGGAAAAGACAAG	molybdate ABC transporter permease subunit
AB395_RS12150	*tonB*	TACGCTTTACTGTCACATCCAAT<br>GCTGCCTGGTCGAGAATT	Periplasmic protein TonB
AB395_RS16720	*sitB*	CTGAAGAAGAATCTCGTCGCCTAT<br>GCCCATCATCACCACATCCT	manganese/iron ABC transporter ATP-binding protein

Table S4. Description of genes in Fig. 4

Gene ID	Gene name	Log_2FC	P-adjust	Gene production
AB395_RS12225	*mtlK*	-0.23	0.4309881	Multiple polyol-specific dehydrogenase (EC 1.1.1.-)
AB395_RS15790	*xylA*	0.01	0.9895518	Xylose isomerase (EC 5.3.1.5)
AB395_RS16435	*frk*	-0.73	0.0093495	ROK family Glucokinase with ambiguous substrate specificity
AB395_RS00575	*frk*	-0.15	0.6709546	Fructokinase (EC 2.7.1.4)
AB395_RS25675	*fbp*	1.61	7.101E-08	Fructose-1,6-bisphosphatase2C type I (EC 3.1.3.11)
AB395_RS10390	*pfp*	0.80	0.0009494	Pyrophosphate-dependent fructose 6-phosphate-1-kinase (EC 2.7.1.90)
AB395_RS32620	*pfp*	4.66	5.244E-61	Tagatose-6-phosphate kinase (EC 2.7.1.144) / 1-phosphofructokinase (EC 2.7.1.56)
AB395_RS15055	*fba*	1.80	1.906E-14	Fructose-bisphosphate aldolase class I (EC 4.1.2.13)
AB395_RS24590	*fba*	-0.45	0.1057599	Fructose-bisphosphate aldolase class II (EC 4.1.2.13)
AB395_RS25690	*fba*	1.92	7.32E-11	Fructose-bisphosphate aldolase class II (EC 4.1.2.13)
AB395_RS32440	*glk*	3.39	4.334E-60	Glucokinase (EC 2.7.1.2)
AB395_RS32660	*glk*	2.82	9.941E-17	Glucokinase like protein
AB395_RS18785	*glk*	-0.55	0.0499549	Glucokinase (EC 2.7.1.2)
AB395_RS00585	*pgi*	-0.29	0.3361126	Glucose-6-phosphate isomerase (EC 5.3.1.9)
AB395_RS01690	*zwf*	-0.48	0.0752833	Glucose-6-phosphate 1-dehydrogenase (EC 1.1.1.49)
AB395_RS01685	*pgl*	-0.35	0.2497178	6-phosphogluconolactonase (EC 3.1.1.31)2C eukaryotic type
AB395_RS01680	*edd*	0.07	0.8659079	Phosphogluconate dehydratase (EC 4.2.1.12)
AB395_RS15725	*eda*	-0.49	0.1408175	4-Hydroxy-2-oxoglutarate aldolase (EC 4.1.3.16) &2-dehydro-3-deoxyphosphogluconate aldolase (EC 4.1.2.14)
AB395_RS08835	*gnd*	-0.28	0.3675601	6-phosphogluconate dehydrogenase2C decarboxylating (EC 1.1.1.44)
AB395_RS25710	*rpe*	1.06	0.0051373	Ribulose-phosphate 3-epimerase (EC 5.1.3.1)
AB395_RS07520	*rpe*	-0.48	0.1355813	Ribulose-phosphate 3-epimerase (EC 5.1.3.1)
AB395_RS08395	*rpi*	-0.59	0.0296624	Ribose 5-phosphate isomerase A (EC 5.3.1.6)
AB395_RS23580	*rpi*	0.07	0.880628	Ribose 5-phosphate isomerase B (EC 5.3.1.6)
AB395_RS32855	*xfp*	4.27	6.022E-69	Xylulose-5-phosphate phosphoketolase (EC 4.1.2.9) & Fructose-6-phosphate phosphoketolase (EC 4.1.2.22)
AB395_RS25360	*tkt*	-0.13	0.7609231	Transketolase2C N-terminal section (EC 2.2.1.1)
AB395_RS25365	*tkt*	-0.08	0.8900902	Transketolase2C C-terminal section (EC 2.2.1.1)
AB395_RS15035	*tkt*	-0.43	0.0896349	Transketolase (EC 2.2.1.1)
AB395_RS25685	*tkt*	1.78	2.497E-12	Transketolase (EC 2.2.1.1)
AB395_RS16790	*tal*	0.18	0.5432929	Transaldolase (EC 2.2.1.2)
AB395_RS11385	*tal*	-0.26	0.6834022	Transaldolase (EC 2.2.1.2)
AB395_RS11300	*prsA*	-0.95	0.0001463	Ribose-phosphate pyrophosphokinase (EC 2.7.6.1)
AB395_RS32630	*prsA*	4.93	1.805E-79	Ribose-phosphate pyrophosphokinase (EC 2.7.6.1)
AB395_RS15040	*gap*	0.12	0.720159	NADPH-dependent glyceraldehyde-3-phosphate dehydrogenase (EC 1.2.1.13) / NAD-dependent glyceraldehyde-3-phosphate dehydrogenase (EC 1.2.1.12)
AB395_RS15050	*pgk*	-0.27	0.372946	Phosphoglycerate kinase (EC 2.7.2.3)

AB395_RS18800	*gpm*	-0.36	0.2381711	Phosphoglycerate mutase (EC 5.4.2.1)
AB395_RS02695	*gpm*	-1.15	3.781E-05	Phosphoglycerate mutase family
AB395_RS05795	*eno*	-0.13	0.7015901	Enolase (EC 4.2.1.11)
AB395_RS15210	*pyk*	-0.69	0.0061332	Pyruvate kinase (EC 2.7.1.40)
AB395_RS32655	*pps*	3.58	9.417E-36	Phosphoenolpyruvate synthase (EC 2.7.9.2)
AB395_RS02910	*pps*	-0.07	0.8134841	Pyruvatephosphate dikinase (EC 2.7.9.1)
AB395_RS05805	*pdh*	0.87	0.000561	Pyruvate dehydrogenase E1 component alpha subunit (EC 1.2.4.1)
AB395_RS05810	*pdh*	0.73	0.0032949	Pyruvate dehydrogenase E1 component beta subunit (EC 1.2.4.1)
AB395_RS32330	*pdh*	2.02	3.244E-17	Pyruvate dehydrogenase E1 component alpha subunit (EC 1.2.4.1)
AB395_RS32335	*pdh*	2.11	2.893E-17	Pyruvate dehydrogenase E1 component beta subunit (EC 1.2.4.1)
AB395_RS18180	*pckA*	1.18	2.524E-08	Phosphoenolpyruvate carboxykinase [ATP] (EC 4.1.1.49)
AB395_RS21690	*gltA*	0.57	0.0359065	Citrate synthase (si) (EC 2.3.3.1)
AB395_RS06120	*gltA*	0.56	0.0318637	Citrate synthase (si) (EC 2.3.3.1)
AB395_RS27600	*acnA*	-0.23	0.4264916	Aconitate hydratase (EC 4.2.1.3)
AB395_RS17375	*acnA*	0.15	0.5658562	Aconitate hydratase (EC 4.2.1.3)
AB395_RS08090	*icd*	-1.19	3.139E-07	Isocitrate dehydrogenase [NADP] (EC 1.1.1.42)
AB395_RS16840	*sucA*	0.38	0.1176081	2-oxoglutarate dehydrogenase E1 component (EC 1.2.4.2)
AB395_RS16835	*sucB*	0.23	0.435535	Dihydrolipoamide succinyltransferase component (E2) of 2-oxoglutarate dehydrogenase complex (EC 2.3.1.61)
AB395_RS05830	*lpd*	0.76	0.0031442	Dihydrolipoamide dehydrogenase of pyruvate dehydrogenase complex (EC 1.8.1.4)
AB395_RS15995	*lpd*	1.67	9.887E-14	Dihydrolipoamide dehydrogenase of branched-chain alpha-keto acid dehydrogenase (EC 1.8.1.4)
AB395_RS16815	*lpd*	0.32	0.2140103	Dihydrolipoamide dehydrogenase of 2-oxoglutarate dehydrogenase (EC 1.8.1.4)
AB395_RS05815	*lpd*	0.68	0.0091526	Dihydrolipoamide acetyltransferase component of pyruvate dehydrogenase complex (EC 2.3.1.12)
AB395_RS32340	*lpd*	1.60	7.382E-10	Dihydrolipoamide acetyltransferase component of pyruvate dehydrogenase complex (EC 2.3.1.12)
AB395_RS15525	*pgm*	1.03	2.422E-05	Phosphoglucomutase (EC 5.4.2.2)
AB395_RS08840	*ccoS*	1.81	0.0010779	Type cbb3 cytochrome oxidase biogenesis protein CcoS involved in heme b insertion
AB395_RS08845	*ccoI*	4.12	1.375E-60	Type cbb3 cytochrome oxidase biogenesis protein CcoI Copper-translocating P-type ATPase (EC 3.6.3.4)
AB395_RS08850	*ccoH*	5.10	1.892E-67	Type cbb3 cytochrome oxidase biogenesis protein CcoH
AB395_RS08855	*ccoG*	5.23	5.028E-79	Type cbb3 cytochrome oxidase biogenesis protein CcoG
AB395_RS08875	*ccoP*	4.13	3.993E-58	Cytochrome c oxidase subunit CcoP (EC 1.9.3.1)
AB395_RS08880	*ccoQ*	3.08	7.577E-10	Cytochrome c oxidase subunit CcoQ (EC 1.9.3.1)
AB395_RS08885	*ccoO*	4.41	2.215E-45	Cytochrome c oxidase subunit CcoO (EC 1.9.3.1)
AB395_RS08890	*ccoN*	5.06	9.709E-89	Cytochrome c oxidase subunit CcoN (EC 1.9.3.1)
AB395_RS04840	*nuoA*	0.41	0.2492021	NADH ubiquinone oxidoreductase chain A (EC 1.6.5.3)
AB395_RS04845	*nuoB*	0.40	0.1284137	NADH-ubiquinone oxidoreductase chain B (EC 1.6.5.3)
AB395_RS04850	*nuoC*	0.41	0.1074975	NADH-ubiquinone oxidoreductase chain C (EC 1.6.5.3)
AB395_RS04855	*nuoD*	0.59	0.0240792	NADH-ubiquinone oxidoreductase chain D (EC 1.6.5.3)

AB395_RS04865	*nuoE*	0.62	0.0088352	NADH-ubiquinone oxidoreductase chain E (EC 1.6.5.3)
AB395_RS04870	*nuoF*	0.41	0.1176949	NADH-ubiquinone oxidoreductase chain F (EC 1.6.5.3)
AB395_RS04885	*nuoH*	0.29	0.3228827	NADH-ubiquinone oxidoreductase chain H (EC 1.6.5.3)
AB395_RS04890	*nuoI*	0.19	0.5246981	NADH-ubiquinone oxidoreductase chain I (EC 1.6.5.3)
AB395_RS04895	*nuoJ*	0.26	0.3385803	NADH-ubiquinone oxidoreductase chain J (EC 1.6.5.3)
AB395_RS04900	*nuoK*	-0.04	0.9198044	NADH-ubiquinone oxidoreductase chain K (EC 1.6.5.3)
AB395_RS04905	*nuoL*	0.01	0.9899626	NADH-ubiquinone oxidoreductase chain L (EC 1.6.5.3)
AB395_RS04910	*nuoM*	-0.02	0.9673037	NADH-ubiquinone oxidoreductase chain M (EC 1.6.5.3)
AB395_RS04915	*nuoN*	0.03	0.9230476	NADH-ubiquinone oxidoreductase chain N (EC 1.6.5.3)
AB395_RS10830	*nuoH*	0.54	0.0223183	NADH-ubiquinone oxidoreductase chain H (EC 1.6.5.3)
AB395_RS10835	*nuoI*	1.27	5.217E-05	NADH-ubiquinone oxidoreductase chain I (EC 1.6.5.3)
AB395_RS10845	*nuoG*	0.94	0.00019	NADH-ubiquinone oxidoreductase chain G (EC 1.6.5.3)
AB395_RS10850	*nuoF*	0.97	0.0019513	NADH-ubiquinone oxidoreductase chain F (EC 1.6.5.3)
AB395_RS10855	*nuoE*	1.57	0.0027839	NADH-ubiquinone oxidoreductase chain E (EC 1.6.5.3)
AB395_RS10860	*nuoD*	1.24	2.233E-06	NADH-ubiquinone oxidoreductase chain D (EC 1.6.5.3)
AB395_RS10865	*nuoC*	1.89	1.602E-07	NADH-ubiquinone oxidoreductase chain C (EC 1.6.5.3)
AB395_RS10870	*nuoB*	2.04	9.417E-09	NADH-ubiquinone oxidoreductase chain B (EC 1.6.5.3)
AB395_RS10875	*nuoA*	1.76	4.578E-07	NADH ubiquinone oxidoreductase chain A (EC 1.6.5.3)
AB395_RS10880	*nuoN*	0.17	0.6633655	NADH-ubiquinone oxidoreductase chain N (EC 1.6.5.3)
AB395_RS10885	*nuoM*	0.20	0.6270663	NADH-ubiquinone oxidoreductase chain M (EC 1.6.5.3)
AB395_RS10900	*mnhD*	0.28	0.5006408	NADH-ubiquinone oxidoreductase chain L (EC 1.6.5.3)
AB395_RS10905	*nuoK*	0.64	0.4893503	NADH-ubiquinone oxidoreductase chain K (EC 1.6.5.3)
AB395_RS10910	*nuoJ*	0.74	0.0071658	NADH-ubiquinone oxidoreductase chain J (EC 1.6.5.3)
AB395_RS23060	*exoP*	1.21	3.337E-07	Succinoglycan biosynthesis transport protein exoP
AB395_RS23065	*exoN*	1.57	5.587E-12	UTP-glucose-1-phosphate uridylyltransferase (EC 2.7.7.9)
AB395_RS00880	*galE*	2.15	1.444E-34	UDP-glucose 4-epimerase (EC 5.1.3.2)
AB395_RS23070	*exoO*	1.22	6.976E-08	Succinoglycan biosynthesis protein ExoO
AB395_RS23075	*exoM*	1.26	9.182E-08	Succinoglycan biosynthesis protein ExoM
AB395_RS23080	*exoA*	1.29	5.612E-09	Succinoglycan biosynthesis protein ExoA
AB395_RS23085	*exoL*	1.54	1.493E-12	Succinoglycan biosynthesis protein ExoL
AB395_RS23090	*exoK*	2.05	1.407E-21	Endo-beta-1,3-1,4 glucanase (EC 3.2.1.73)
AB395_RS23105	*exoI*	0.59	0.027931	putative periplasmic protein
AB395_RS23110	*exoU*	1.44	1.128E-11	Succinoglycan biosynthesis protein ExoU
AB395_RS23115	*exoX*	1.22	0.0073245	Exopolysaccharide production repressor protein
AB395_RS23120	*exoY*	1.31	1.316E-09	Exopolysaccharide biosynthesis protein
AB395_RS24220	*nrtC*	2.46	5.981E-17	Nitrate ABC transporter ATP-binding protein
AB395_RS24225	*nrtB*	2.11	5.959E-10	Bicarbonate transport system permease protein
AB395_RS24230	*nrtA*	2.18	8.217E-12	Nitrate ABC transporter nitrate-binding protein
AB395_RS21200	*urtA*	2.21	2.89E-17	urea ABC transporter substrate-binding protein
AB395_RS21205	*urtB*	2.61	3.214E-25	urea ABC transporter permease subunit UrtB
AB395_RS21210	*urtC*	2.97	3.983E-39	urea ABC transporter permease subunit UrtC
AB395_RS21215	*urtD*	3.07	4.731E-31	urea ABC transporter ATP-binding protein UrtD
AB395_RS21220	*urtE*	3.42	4.016E-52	urea ABC transporter ATP-binding subunit UrtE

AB395_RS04560	*napE*	3.34	9.481E-27	Periplasmic nitrate reductase component NapE
AB395_RS04565	*napF*	2.24	2.215E-20	Ferredoxin-type protein NapF (periplasmic nitrate reductase)
AB395_RS04570	*napD*	1.80	0.0046529	Periplasmic nitrate reductase component NapD
AB395_RS04575	*napA*	2.02	4.6E-27	Periplasmic nitrate reductase precursor (EC 1.7.99.4)
AB395_RS04580	*napB*	1.88	6.78E-12	Nitrate reductase cytochrome c550-type subunit
AB395_RS04585	*napC*	1.53	9.234E-11	Cytochrome c-type protein NapC
AB395_RS26435	*nasA*	0.81	0.0197989	Assimilatory nitrate reductase large subunit (EC:1.7.99.4)
AB395_RS04535	*nnrS*	3.56	2.219E-32	NnrS protein involved in response to NO
AB395_RS04540	*nirK*	3.77	1.707E-59	Copper-containing nitrite reductase (EC 1.7.2.1)
AB395_RS04545	*nirV*	2.32	1.074E-18	Nitrite reductase accessory protein NirV
AB395_RS04550	*nnrR*	0.18	0.5852129	Nitric oxide -responding transcriptional regulator NnrR
AB395_RS26425	*nirB*	-0.54	0.4524857	Nitrite reductase [NAD(P)H] large subunit (EC 1.7.1.4)
AB395_RS26430	*nirD*	0.84	0.0165051	Nitrite reductase [NAD(P)H] small subunit (EC 1.7.1.4)
AB395_RS08065	*glsA*	-1.36	2.421E-05	Glutaminase (EC 3.5.1.2)
AB395_RS21835	*glnA*	1.39	4.45E-10	Glutamine synthetase type II (EC 6.3.1.2)
AB395_RS27410	*glnA*	-0.24	0.5860045	Glutamine synthetase type I (EC 6.3.1.2)
AB395_RS01700	*glnA*	0.49	0.0296624	glutamine synthetase family protein
AB395_RS07005	*glnA*	-0.11	0.7735058	Glutamine synthetase type I (EC 6.3.1.2)
AB395_RS10505	*glnA*	0.51	0.0701031	glutamine synthetase family protein
AB395_RS26035	*gdhA*	0.42	0.2775176	glutamate dehydrogenase
AB395_RS12345	*ureG*	1.06	8.612E-05	Urease accessory protein UreG
AB395_RS12350	*ureF*	-0.17	0.6804141	Urease accessory protein UreF
AB395_RS12355	*ureE*	0.29	0.3221612	Urease accessory protein UreE

Table S5. Strains, plasmids and primers used in this study

	Relevant characteristics	Note
**Strains**		
E. coli DH5α	F-φ80 lac ZΔM15 Δ(lacZYA-arg F) U169 endA1 recA1 hsdR17(rk-,mk+) supE44λ- thi -1 gyrA96 relA1 phoA	Biomed
S. fredii CCBAU45436	*S. fredii* CCBAU45436 wild type, NA^r, TMP^r	(1)
Δ*rsh*	*S. fredii* CCBAU45436 *rsh* in-frame deletion, NA^r, TMP^r	This work
**Plasmids**		
pJQ200SK	suicide plasmid with sacB selectable marker, Gm^r	(2)
pRK2013	Helper plasmid, IncP, Tra^+, Mob^+, ColE1, Km^r	(3)
**Primers for *rsh* deletion**		
rsh-UF	ATCGAATTCCTGCAGCCCTCCGGTTTCGTGTCGTTAATC	
rsh-UR	AATTCCTTGACTCGTATTGGCGCATCATTC	
rsh-DF	CCAATACGAGTCAAGGAATTGCCGAGCATC	
rsh-DR	AGAACTAGTGGATCCCCCTTGATGGGCGCAAGATATGC	
rsh-WF	CATCGCGGAAATGTCGTATTC	
rsh-WR	CCGTCATCGCATCTTGAAAC	

References

1. Zhang YM, Li Y, Jr., Chen WF, Wang ET, Tian CF, Li QQ, Zhang YZ, Sui XH, Chen WX. 2011. Biodiversity and biogeography of rhizobia associated with soybean plants grown in the North China Plain. Appl Environ Microbiol 77:6331-42.
2. Quandt J, Hynes MF. 1993. Versatile suicide vectors which allow direct selection for gene replacement in Gram-negative bacteria. Gene 127:15-21.
3. Figurski DH, Helinski DR. 1979. Replication of an origin-containing derivative of plasmid RK2 dependent on a plasmid function provided in trans. Proc Natl Acad Sci U S A 76:1648-52.
